# Antibacterial activity of propylene glycol against *Staphylococcus aureus* and *Staphylococcus epidermidis* in neutral and mild acidic conditions

**DOI:** 10.1093/jac/dkaf153

**Published:** 2025-05-26

**Authors:** Nadiia Mosiichuk, Gabriela Enggren, Zita Lopes Da Silva, Anna Karin Morén, Henri Hansson, Johan Engblom, Tautgirdas Ruzgas

**Affiliations:** Department of Biomedical Science, Faculty of Health and Society, Malmö University, Malmö, Sweden; Biofilms Research Centre for Biointerfaces, Malmö University, Malmö, Sweden; Department of Biomedical Science, Faculty of Health and Society, Malmö University, Malmö, Sweden; Biofilms Research Centre for Biointerfaces, Malmö University, Malmö, Sweden; Biofilms Research Centre for Biointerfaces, Malmö University, Malmö, Sweden; Department of Oral Biology, Faculty of Odontology, Malmö University, Malmö, Sweden; Galenica AB, Medeon Science Park, Malmö, Sweden; Galenica AB, Medeon Science Park, Malmö, Sweden; Department of Biomedical Science, Faculty of Health and Society, Malmö University, Malmö, Sweden; Biofilms Research Centre for Biointerfaces, Malmö University, Malmö, Sweden; Department of Biomedical Science, Faculty of Health and Society, Malmö University, Malmö, Sweden; Biofilms Research Centre for Biointerfaces, Malmö University, Malmö, Sweden

## Abstract

**Background:**

*Staphylococcus aureus* and *Staphylococcus epidermidis* normally coexist on the skin of healthy individuals. Their ratio and pathogenicity, however, change with different skin diseases and thus, the ability to control bacterial growth on the skin is important. Propylene glycol (PG), a widely used component in topical formulations, has been proved to have antimicrobial activity.

**Objectives:**

To investigate the concentration-dependent antimicrobial properties of PG against *S. aureus* and *S. epidermidis* skin isolates in mono- and co-culture at neutral (pH 7.4) and mildly acidic (pH 5) conditions.

**Results and discussion:**

The minimum inhibitory concentration of PG was 12.5% at both pH 5 and pH 7.4 for the selected bacterial strains. The viability of *S. aureus* exposed to 12.5% PG was lower at pH 5 than at pH 7.4, and post-treatment regrowth of *S. aureus* occurred slowly at acidic pH. When both bacterial strains were incubated in media containing 12.5% PG at pH 5 for 48 hours, *S. epidermidis* retained significantly higher viability, while at pH 7.4, the results were opposite.

**Conclusions:**

Under the acidic conditions of healthy skin, pathogenic *S. aureus* is more suppressed by 12.5% PG than commensal *S. epidermidis*.

## Introduction

Commensal staphylococcal species are among the most abundant colonizers of human skin, with *Staphylococcus epidermidis* and *Staphylococcus hominis* being the most frequently isolated from all skin sites.^1^  *Staphylococcus aureus* is the most problematic pathogen of the genus, causing numerous acute and chronic infections, although it can be found on the skin of 10%–20% of healthy individuals.^2^ Many skin diseases, particularly acne, atopic dermatitis (AD) and chronic wounds, are associated with alterations in the ratio of commensal and pathogenic staphylococcal species. For example, *S. aureus* can be 100 times more abundant in AD skin compared to normal healthy skin.^3^ Among the factors that contribute to growth and propagation of pathogenic bacteria during diseases is skin pH. The pH of healthy skin across different body parts ranges from 4.1 to 5.8, whereas in under skin eczema and AD, the pH is 0.1–0.9 units higher than that of healthy skin.^4^

The frequent use of topical antibiotics for staphylococcal infections results in antimicrobial resistance. Therefore, there is a need for research into alternative antibacterial therapies that may serve as topical agents for the prevention and treatment of skin diseases at an early stage.

Propylene glycol (PG) is a very abundant component in many topical formulations for daily skin care, as well as for the treatment of skin lesions.^5,6^ Its antimicrobial action was discovered many years ago,^7–10^ but there are still gaps in knowledge regarding the bactericidal concentration for some microbial strains. The antimicrobial efficacy of PG can vary depending on factors such as concentration, pH, formulation and the specific microorganisms targeted.^7–10^

In the present study we investigated the concentration-dependent antimicrobial properties of PG against *S. aureus* and *S. epidermidis* in mono- and co-culture at neutral and mild acidic conditions.

## Materials and methods

*S. aureus ZB* and *S. epidermidis ZA* strains were isolated from the dorsum, palm and inner forearm surface of a healthy person’s hand. Identification was carried out using 16S rRNA gene sequencing at the Clinical Microbiology Laboratory, Region Skåne (Lund, Sweden). Bacterial strains were preserved as 10% skim milk stocks at −20°C.

The MICs of PG were determined using the broth microdilution method at pH 7.4 and 5. The susceptibility of isolates to ampicillin and gentamicin were determined as previously described.^11^ The detailed protocol is given in the Supplementary Material (available as Supplementary data at *JAC* Online).

To assess the viability of bacteria in mono- and co-culture, overnight individual cultures of *S. aureus* and *S. epidermidis* were diluted to 10^5^ cfu/mL. The co-culture was prepared by taking a 1:1 ratio of *S. aureus* and *S. epidermidis* and spiked with 12.5% PG, then incubated for 48 hours at 37°C. Control samples contained the same volume of sterile distilled water instead of PG. The cultures were sampled at 24- and 48-hour time points, and the numbers of bacteria were estimated by cfu enumeration on BHI agar media. The detailed protocol is given in the Supplementary Material.

To investigate the effects of pH on regrowth of bacteria after PG treatment, the co-cultures of *S. aureus* and *S. epidermidis* were incubated in MH broth with 12.5% PG and pH 5 or 7.4 for 24 h. Then bacterial cells were pelleted, resuspended in fresh MH broth with the same pH and incubated for another 24 h at 37°C. Control samples underwent the same procedure with PG substituted by water. Cultures were sampled at the indicated time points, and the numbers of bacteria were estimated by cfu enumeration on BHI agar media.

All values are expressed as the mean ± SEM. The data were analysed using GraphPad Prism v.10 software (San Diego, CA) and compared using an unpaired Student’s *t*-test. A *P* value of <0.05 was considered statistically significant.

## Results and discussion

### *Effect of PG on growth of* S. aureus *and* S. epidermidis *under different pH*

The antimicrobial action of PG has been reported >50 years ago, however, without clear data concerning MIC of PG on staphylococcal species. In this study we clearly demonstrated that MIC of PG for both *Staphylococcus* strains is 12.5% at pH 5 and 7.4 (Figures S1 and S2, available as Supplementary data). However, at pH 5, the growth of *S. aureus* was almost completely inhibited at 6.25% PG.

Both isolates demonstrated the same susceptibility to gentamicin (MIC = 0.031 mg/L), but slightly different to ampicillin (MIC = 0.25 mg/L for *S. aureus*, MIC = 0.5 mg/L for *S. epidermidis*, pH = 7.4) (Figure S3, Supplementary data).

**Figure 1.**
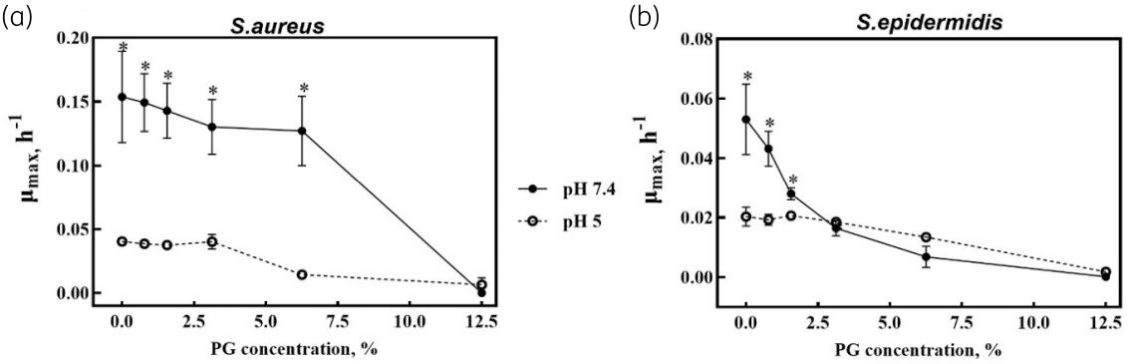
Effect of different concentrations (%) of PG on the maximum specific growth rates (μ) of *S. aureus* (a) and *S. epidermidis* (b) in MH broth at pH 7.4 and 5. μ values were obtained by fitting growth curves using a sigmoidal empirical growth model.^13^ Error bars represent SEM of at least three independent experiments that were performed in triplicate (three independent wells). Asterisks indicate a significant difference between values at the same point at pH 5 and 7.4, *P* < 0.05 (unpaired *t*-test).

The pH value of the medium had a significant effect on bacterial growth with and without PG (control). The maximal growth rate μ of *S. aureus* was substantially lower at pH 5 than at pH 7.4 in control and at low PG concentrations (≤6.25%, Figure 1a). The growth of *S. epidermidis* at pH 5 showed significantly lower μ at 0–1.56% PG concentrations as compared with those at pH 7.4 (Figure 1b). The lag time increased with the increase of PG concentration in both bacterial strains at both pH studied (Figures S1c and S2c). Our results are in agreement with those obtained by Iyer and co-authors,^12^ who showed that *S. aureus* had lower growth rate when exposed to acidic pH, while *S. epidermidis* grown similarly at pH within the range of 5–7.

### *Bactericidal effect of 12.5% PG on* S. aureus *and* S. epidermidis *in mono and co-culture*

**Figure 2.**
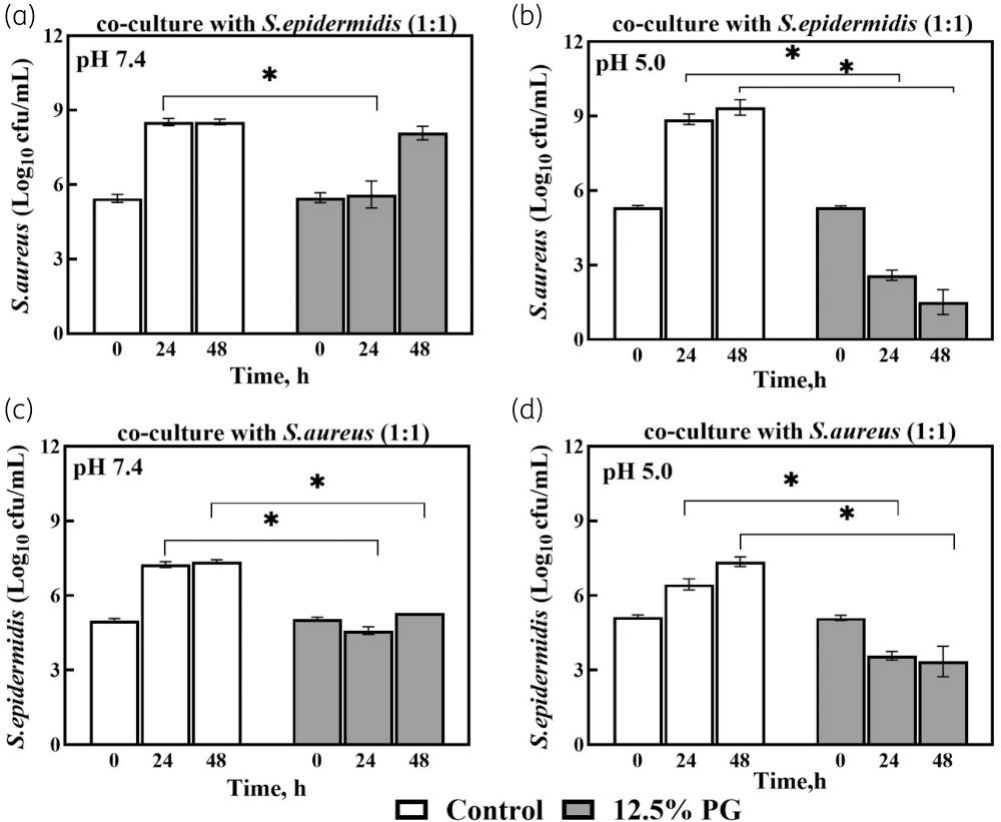
Effect of 12.5% PG on viability of *S. aureus* and *S. epidermidis* after 24 and 48 h of incubation in the MH broth with different pHs in co-culture. The initial concentration of bacterial cells in co-culture was 10^5^ cfu/mL and the starting ratio of *S. aureus* to *S. epidermidis* was 1:1. Cultures were incubated for 48 h in total (37°C), but for the viability test sampled at the indicated 0-, 24- and 48-h time points. Data are presented as mean ± SEM of at least three independent experiments. *Significantly differs from respective control value at the same time point, *P* < 0.05 (unpaired *t*-test).

*S. epidermidis* can restrict the growth of *S. aureus* through production of antimicrobial molecules.^14–17^ Some species of bacteria can alter the antimicrobial susceptibility of others *in vitro* and *in vivo*.^18–20^ Thus, we hypothesized that the interaction of *S. aureus* and *S. epidermidis* in co-culture could change individual susceptibility of these bacteria to PG. Additionally, we were interested to see whether pH of the environment has an impact on this interaction. As can be seen from Figure 2(a and b) the bactericidal activity of 12.5% PG against *S. aureus* in co-culture was stronger at pH 5 than at pH 7.4. At pH 7.4, treatment with PG resulted in significant reduction in *S. aureus* viability after 24 h, but after 48 h it did not differ from the control (Figure 2a). In contrast, at pH 5, *S. aureus* viability declined significantly with increasing incubation time (Figure 2b).

At both pH values, the viable cell concentration of *S. epidermidis* in co-culture was reduced by 30%–50% after 24 and 48 h of treatment with 12.5% PG, compared to the corresponding controls (Figure 2c and d). The results observed in the co-culture of both bacteria were similar to those in monoculture (Figure S4, Supplementary data), indicating no interaction between commensal *S. epidermidis* and pathogenic *S. aureus* in these experiments.

When both bacterial strains were grown together in MH broth at pH 7.4 with or without PG, *S. aureus* tended to dominate the culture and suppress *S. epidermidis* viability in the system (at 24–48 h). Similarly, at pH 5 without PG, *S. aureus* viable cell concentration reached ∼10^9^ cfu/mL, whereas *S. epidermidis* reached just ∼10^7^ cfu/mL by 48 h in the mixed bacteria culture. By contrast, in the presence of 12.5% PG and pH 5, *S. epidermidis* significantly dominated in the co-culture after 48 h of incubation (Figure S5a, Supplementary data). The viable cell count of *S. epidermidis* was ∼10^3^ cfu/mL, whereas *S. aureus* was just ∼10^1^ cfu/mL. These results indicate that pH of the environment has influence on the bacteria’s susceptibility to PG and at pH 5 the viability of *S. aureus* after treatment with 12.5% PG was lower than *S. epidermidis*. The change of pH in the growth media was also observed and is presented in Supplementary Figure S5(b).

### Post-treatment regrowth of bacteria at different pH

At pH 7.4, *S. aureus* rapidly regrew after removal of PG and reached the control level by 24 h. At the same time, at pH 5, the post-treatment regrowth of bacteria occurred slowly and after 24 h the concentration of viable cell was still significantly lower compared with the control value. The post-treatment regrowth of *S. epidermidis* showed a similar trend in both pHs, and 24 h after removal of PG the viable cell count was significantly lower than that in corresponding controls (Figure S6, Supplementary data).

### Conclusions

In this study, we showed that the MIC of PG for *S. aureus* and *S. epidermidis* skin isolates is 12.5% at pH 7.4 and 5. However, at pH 5, the growth of *S. aureus* was almost completely inhibited at 6.25% PG. The bactericidal activity of PG against *S. aureus* was stronger at pH 5 than at pH 7.4, and the post-treatment regrowth of *S. aureus* occurred slowly at the lower pH. The results observed in the co-culture of both bacteria were similar to those in monoculture, indicating no interaction between commensal *S. epidermidis* and pathogenic *S. aureus* in these experiments. Taken together, we can conclude that at pH 5, which corresponds to the healthy skin pH, pathogenic *S. aureus* is more sensitive to PG than commensal *S. epidermidis*. However, further *in vivo* studies are needed to validate this finding in clinical settings.

## Supplementary Material

dkaf153_Supplementary_Data

## Supplementary data

Figures S1 to S6 are available as Supplementary data at *JAC* Online.
