# Supplementary material for: Antibacterial activity of propylene glycol against Staphylococcus aureus and Staphylococcus epidermidis in neutral and mild acidic conditions
Source: J Antimicrob Chemother. 2025 May 26;80(7):1947–50. doi: 10.1093/jac/dkaf153 (PMC12209790; doi:10.1093/jac/dkaf153)
Supplement: dkaf153_Supplementary_Data [file dkaf153_supplementary_data.docx]

**Supplementary Material**

**Supplementary Materials and Methods**

***Bacteria cultivation***

*S. aureus* and *S. epidermidis* were streaked on plates with Brain Heart Infusion (BHI) agar (MilliporeSigma, Burlington, MA, USA) and grown in 5% carbon dioxide (CO_2_) at 37 °C for 24 h. For each strain, 5 single colonies were transferred to a liquid medium of BHI broth (MilliporeSigma, Burlington, MA, USA) with pH either 5.0 or 7.4 and incubated overnight (17-18 h) at 37°C and 5% CO_2_. pH of the liquid mediums was checked with pH meter (827 pH lab, Metroholm, Zofingen, Switzerland) and adjusted to 5.0 with 3 M hydrochloric acid before autoclaving. 1 mL of the overnight bacterial cells suspension was pelleted at 3000 rpm for 10 min and resuspended in Mueller- Hinton (MH) broth (MilliporeSigma, Burlington, MA, USA) of respective pH to get the optical density 0.07 at 600 nm. Subsequently, the individual bacterial suspensions were diluted with MH broth (pH 5.0 or 7.4, respectively) by 25-fold for *S. aureus* and by 10-fold for *S. epidermidis*. The final concentration of each bacterium in the culture was ∼10^5^ cfu/mL, which was confirmed by cfu enumeration.

***Colony forming assay***

The bacterial suspension was serially diluted 1:10^x^ in sterile PBS buffer (pH 7.4) and 100 µL of each sample was plated onto a BHI agar plate. Plates were incubated at 37°C for 24 h with following incubation at room temperature for 24 h for the final enumeration of viable *S. aureus* and *S. epidermidis* in mono- or co-culture.

***Determination of the Bacterial Growth Inhibition***

Propylene glycol (≥99.5%, Cat. N 398039, Sigma-Aldrich, USA) was serially diluted with MH broth (pH 7.4 or 5) two-fold across the wells of 96-well sterile Greiner flat-bottomed plates (Greiner Bio-One GmbH, Germany). Separate bacterial suspensions of *S. aureus* or *S. epidermidis* with a concentration of approximately 10^5^ cfu/mL were used to inoculate the medium in the wells. Each well contained 100 µL of inoculum and 100 µL of MH broth of appropriate pH with propylene glycol in the final concentrations ranging from 50 to 0.098 % (v:v). Each negative control (blank) well contained 200 µL of MH broth. Each positive control well contained 100 µL of inoculum and 100 µL of MH broth. Each of the combinations was done in triplicate. After inoculation, microplates were covered with a sterile Breathe-Easy® gas permeable sealing membrane (Diversified Biotech, USA) and incubated for 24-48 hours at 37˚C in the SPECTROstar Nano plate reader (BMG LABTECH, Germany). Absorbance was recorded at 600 nm (OD600) every 2 h with a 30 s double orbital shaking (300 rpm) prior to each absorbance reading. At the end of the measurement bacterial suspension from positive control wells was diluted and plated to check purity of the culture. Each growth curve was conducted in triplicate and independently repeated 3-6 times.

***Antibiotic susceptibility test***

Susceptibility of both skin isolates to ampicillin (Sigma-Aldrich, USA) and gentamicin (Gibco™) were determined by the microdilution method in 96-well microtiter plates according to EUCAST guidelines as described previously.^1^ Briefly, overnight cultures were diluted to 10^5^ cfu/mL in MH broth (pH 7.4). Antimicrobial agents were subjected to serial twofold dilution in MH broth and equally mixed with bacterial suspensions in 96-well microtiter plates. The 96-well microtiter plates were incubated at 37°C for 24 h. The lowest concentration that inhibited bacterial growth at 37°C after 24 h was considered the MIC. All assays were repeated two- three times.

***Bacteria viability determination in mono- and co-culture***

The effect of the lowest concentration of PG that completely reduced bacterial cell growth (MIC) on cell viability was studied at each tested pH condition. Separate bacterial suspension of *S. aureus* (∼5×10^5^ cfu/mL) and *S. epidermidis* (∼5×10^5^ cfu/mL) in the MH broth with pH 7.4 or 5 were prepared as described above. Then 8.75 mL of separate suspensions was mixed with 1.25 mL of PG in a 50 mL sterile Falcon® tubes (VWR, USA) to the final concentration of PG 12.5% (v/v). The control samples contained the same amount of sterile distilled water instead of PG. For co-culture experiments, suspensions of *S. aureus* (∼5×10^5^ cfu/mL) and *S. epidermidis* (∼5×10^5^ cfu/mL) with the same pH were mixed (1:1, v/v) in the same volume and PG was added to the final concentration of 12.5% (v/v). The control tube contained the same amount of sterile distilled water instead of PG. Tubes were incubated for 48 h at 37°C and 5% CO2 and 100 µL samples were drawn at 0 (after mixing with PG), 24 and 48 h of incubation, serially diluted with PBS (1:10^0^-1:10^8^) and plated onto BHI agar plate for cfu counting. pH measurements were done with a pH meter (827 pH lab, Metroholm, Zofingen, Switzerland) at the same intervals described above. All experiments were independently repeated at least three times.

***Determination of post-treatment regrowth of bacteria***

The regrowth of bacteria in co-culture after removing PG was studied at different pH. Briefly, suspensions of *S. aureus* (10^5^ cfu/mL) and *S. epidermidis* (10^5^ cfu/mL) with the same pH were mixed (1:1, v/v) and PG was added to the final concentration of 12.5% (v/v). The control tube contained the same amount of sterile distilled water instead of PG. Tubes were incubated for 24 h at 37°C and 5% CO2. Bacteria viability was assessed at 0 h (after mixing with PG), after 24 h of incubation with 12.5% PG via cfu counting. Then suspension was centrifuged at 3000 rpm for 10 min, medium with PG was removed and bacterial pellet was resuspended in fresh MH broth (pH 5.0 or 7.4, respectively). Control tubes underwent the same procedure. Tubes were incubated for 24 h at 37°C and 5% CO2. Bacterial counts were determined at 2, 6 and 24 h time after removing PG. All experiments were performed at least in triplicate.

***Statistical Analysis***

All experiments were performed independently on at least three separate occasions. All values are expressed as the mean ± standard error of mean (SEM). Curve-fitting analyses were performed by using MATLAB software (MathWorks, Inc). The growth curves were fitted using sigmoidal empirical growth model^2^ to derive the maximum specific growth rates (μ) and lag time (λ). All other data were analyzed using GraphPad Prism 10 software (San Diego, CA) and compared using an unpaired Student's t-test. A *p-*value of less than 0.05 was considered statistically significant.

**Supplementary Figures**

**
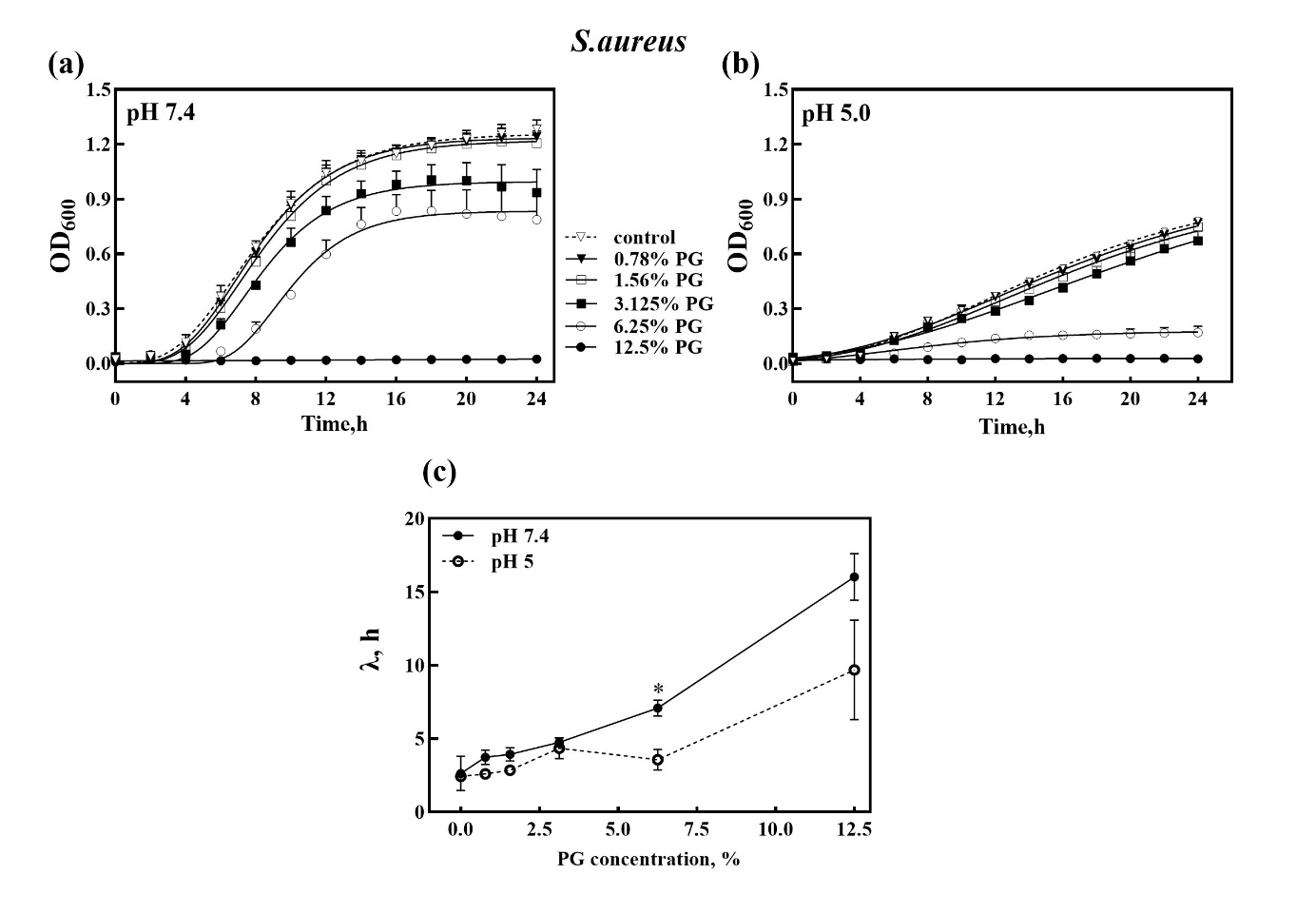
**

**Figure S1.** Growth curves of *S. aureus* at different concentrations (%) of propylene glycol (PG) in MH broth at pH 7.4 (a) and 5(b). Growth curves were fitted using sigmoidal empirical growth model^2^ to derive lag time (λ). Figure (c) shows the dependencies of lag time λ on PG concentration. Error bars represent standard error of the means (SEM) of at least 3 independent experiments that were performed in triplicate (three independent wells). At the figure (c) asterisks indicate significant difference between values at the same point at pH 5 and 7.4, *p<0.05* (unpaired t-test).

**
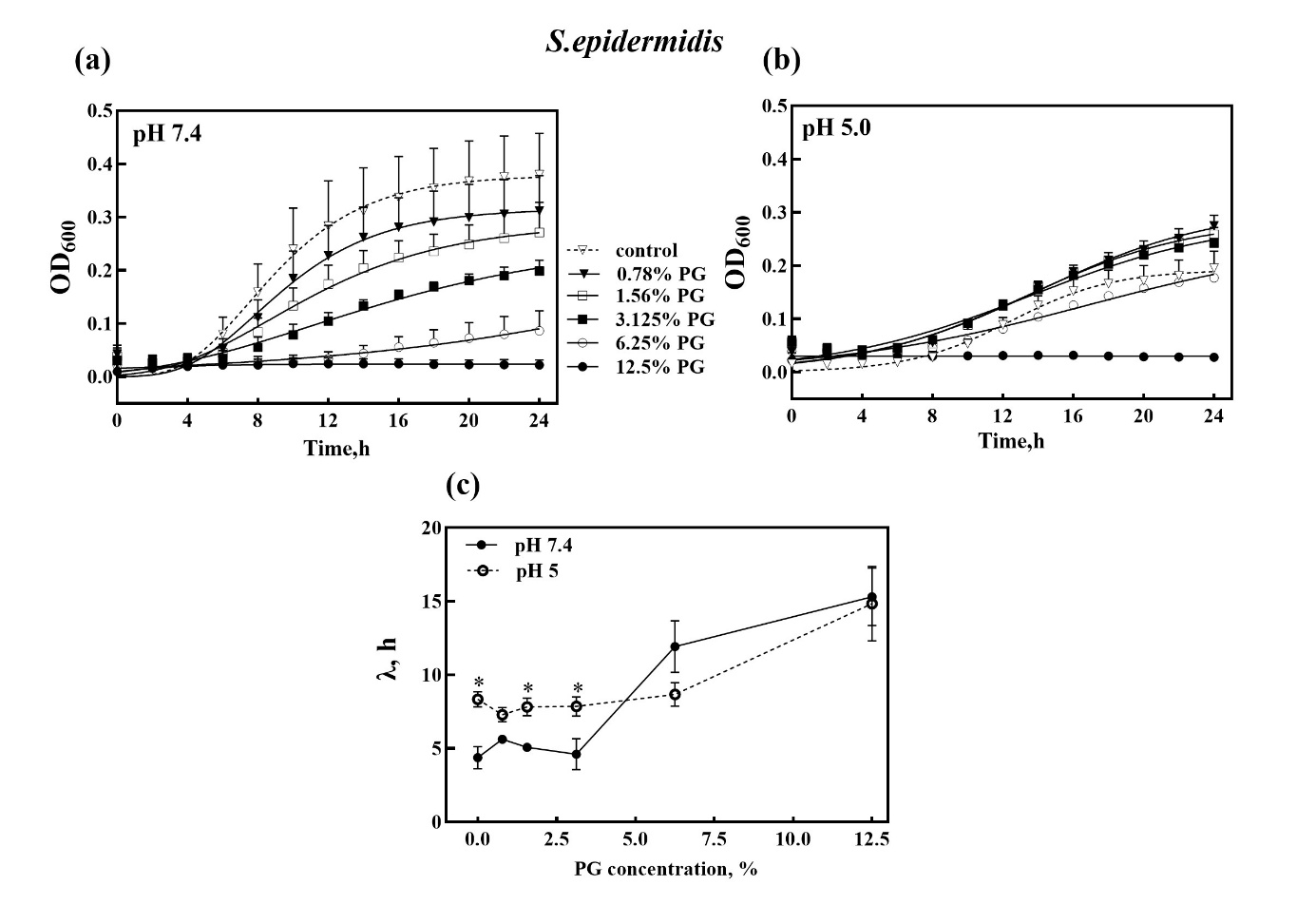
**

**Figure S2.** Growth curves of *S. epidermidis* at different concentrations (%) of propylene glycol (PG) in MH broth at pH 7.4 (a) and 5(b). Growth curves were fitted using sigmoidal empirical growth model^2^ to derive the lag time (λ). Figure (c) shows the dependencies of lag time λ on PG concentration. Error bars represent standard errors of the means (SEM) of at least 3 independent experiments that were performed in triplicate (three independent wells). At the figure (c) asterisks indicate significant difference between values at the same point at pH 5 and 7.4, *p<0.05* (unpaired t-test).

***
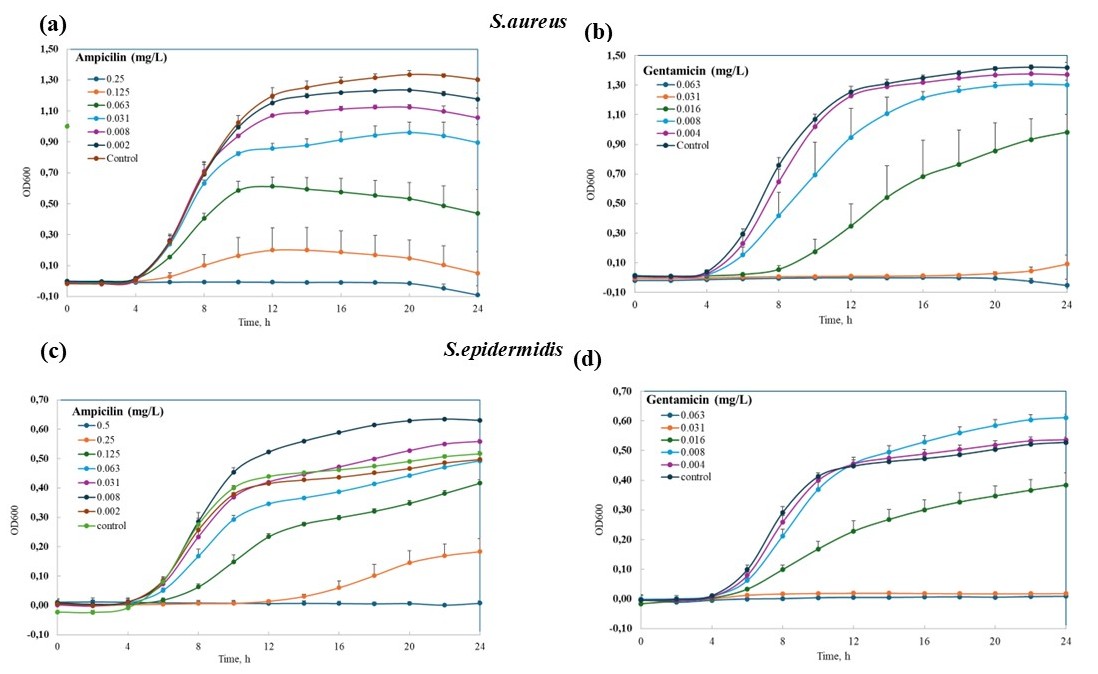
***

**Figure S3.** Growth curves of *S. aureus* and *S. epidermidis* at different concentrations of ampicillin and gentamicin in MH broth at pH 7.4. Error bars represent standard error of the means (SEM) of 2-3 independent experiments that were performed in triplicate (three independent wells).

***
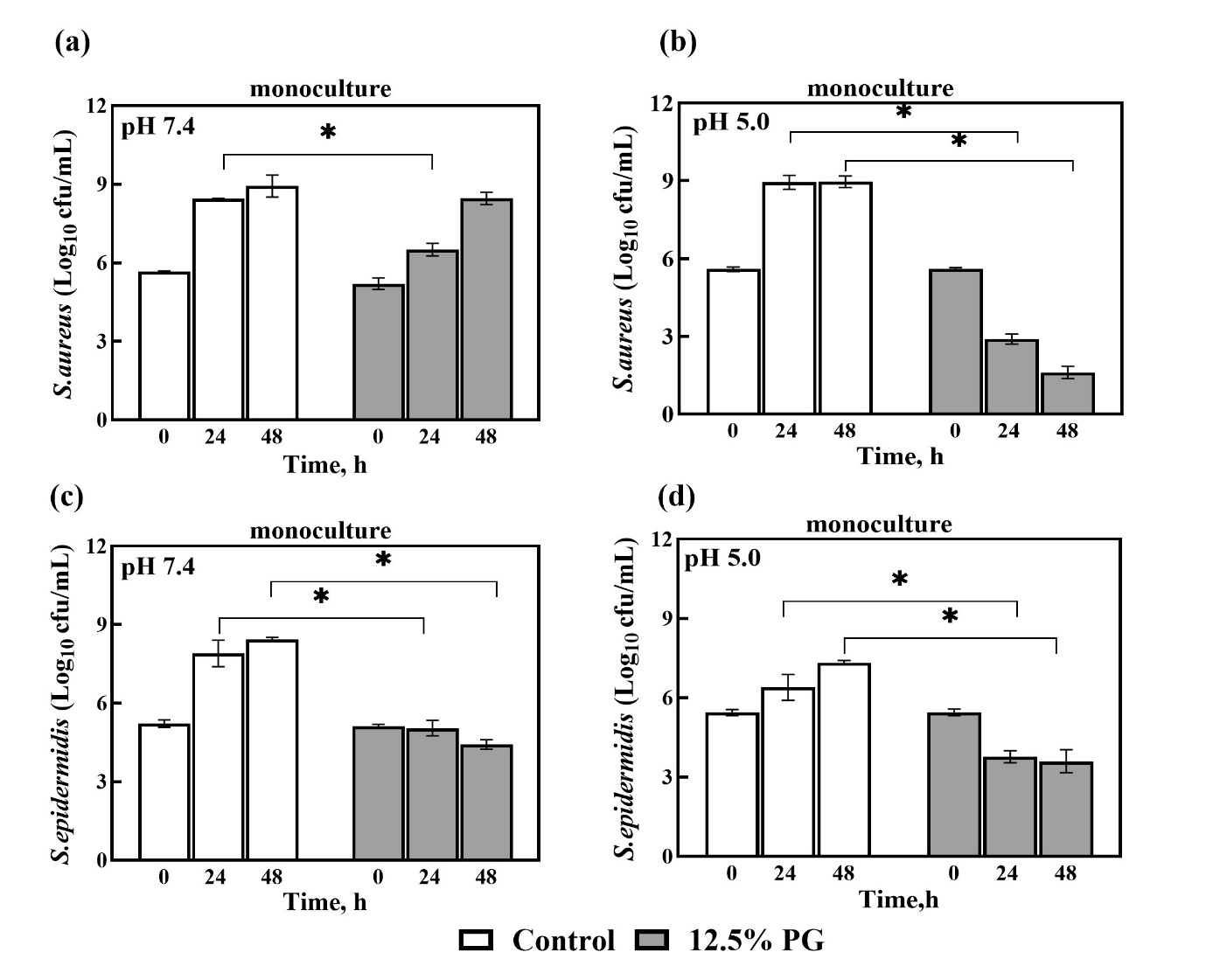
***

**Figure S4.** Effect of 12.5% propylene glycol (PG) on viability of *S. aureus* and *S. epidermidis* after 24 and 48 h incubation in the MH broth with different pH in monoculture. The initial concentration of bacterial cells in monoculture was 10^5^ cfu/mL. Cultures were incubated for 48 h total (37°C), but for viability test sampled at the indicated 0, 24 and 48 h time points. Data are presented as mean ± SEM of at least 3 independent experiments. *Significantly differ from respective control value at the same time point, p<0.05 (unpair t-test).


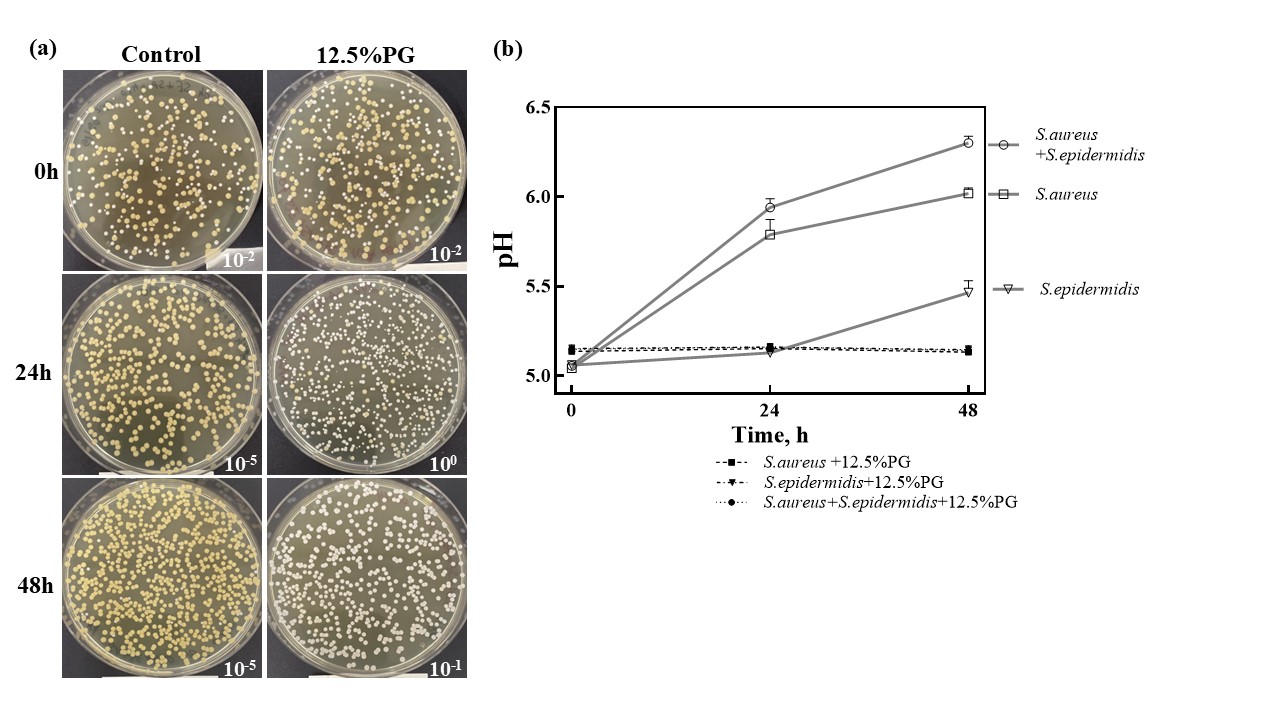


**Figure S5.** (a)The colonies of *S. aureus* (yellow) and *S. epidermidis* (white) on BHI agar plates. The co-cultures had a starting ratio of *S. aureus* to *S. epidermidis* cfu of 1:1 in MH broth (pH 5.0). The mixture was incubated with or without 12.5% PG for 48 hours. Culture suspensions were sampled at the indicated time points (0, 24, and 48 hours), serially diluted 10^0^-10^-5^ in PBS buffer and plated on BHI agar for cfu enumeration. A representative picture from one of the experiments is shown. (b) Change of pH in MH broth inoculated with *S. aureus* and *S. epidermidis* alone and in combination (1:1) during 48 h of incubation with or without 12.5% PG at 37 °C (pH of uninoculated MH: 5). Measurements of pH were performed in the aliquots sampled at the time of initial inoculation (0 h) and after 24 and 48 h of incubation at 37 °C. Data are presented as mean ± SEM of five independent experiments.

***
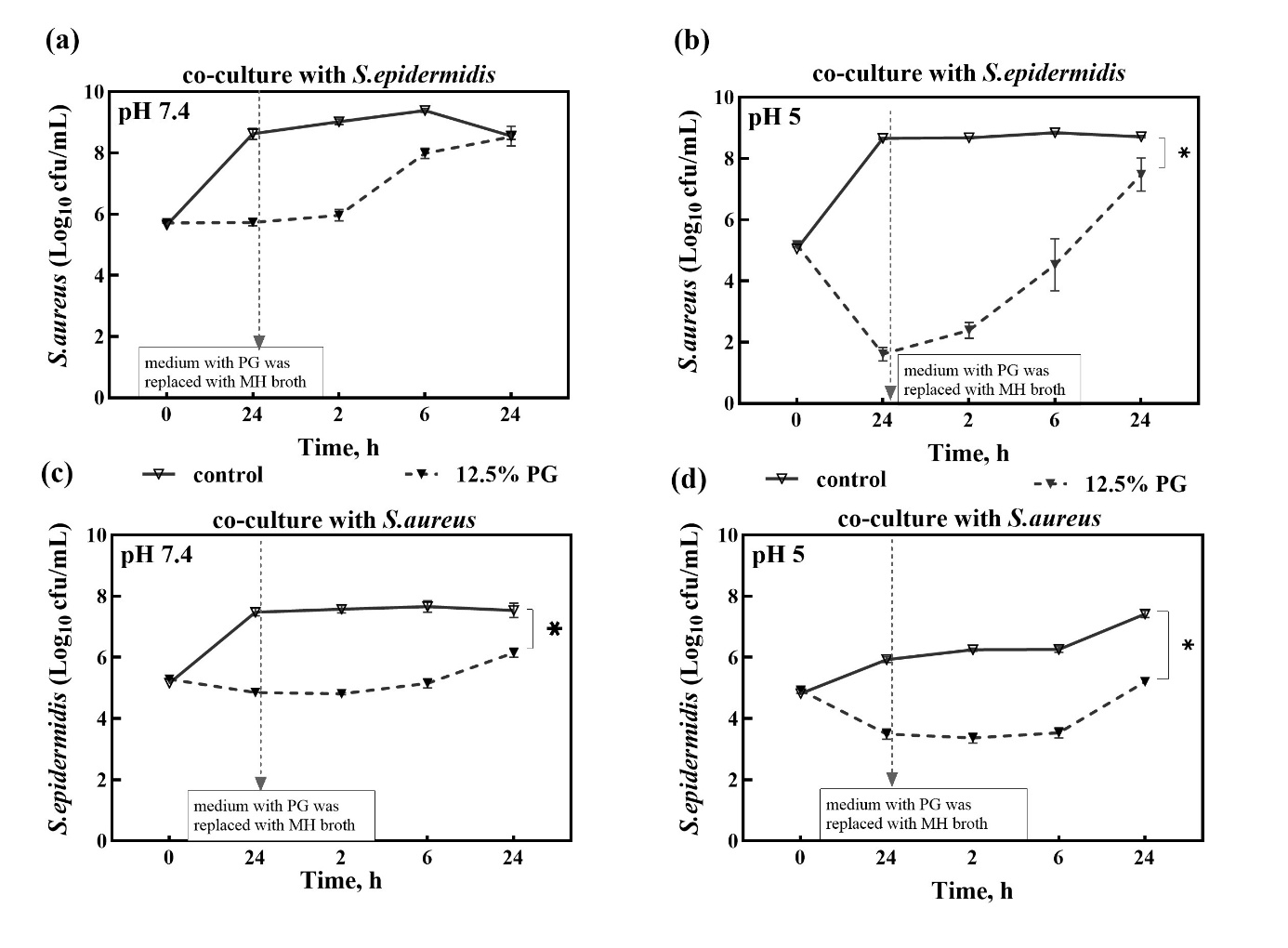
***

**Figure S6.** Post-treatment regrowth of *S. aureus* and *S. epidermidis* after incubation in co-culture with or without 12.5% PG at pH 5 or 7.4. The starting ratio of *S. aureus* to *S. epidermidis* in co-culture was 1:1. The values represent the mean of viable bacterial count per mL (CFU/mL) at 0 h (immediately after mixing) and 24h after PG treatment, as well as 2, 6 and 24 h after replacement of the media. *Significantly differ from respective control value at the same time point, p<0.05 (unpair t-test). Data are presented as mean ± SEM of 2-5 independent experiments.

**References**

1. European Committee for Antimicrobial Susceptibility Testing (EUCAST) of the European Society of Clinical Microbiology and Infectious Diseases (ESCMID). Determination of minimum inhibitory concentrations (MICs) of antibacterial agents by broth dilution. *Clinical Microbiology and Infection*, 2003; **9**:ix-xv.
2. Longhi DA, Dalcanton F, Aragão GMFD et al. Microbial growth models: A general mathematical approach to obtain μ max and λ parameters from sigmoidal empirical primary models. *Braz J Chem Eng* 2017; **34**: 369**-**75. https://doi.org/[10.1590/0104-6632.20170342s20150533](https://doi.org/10.1590/0104-6632.20170342s20150533)
